# Age Influences Likelihood of Pancreatic Cancer Treatment, but not Outcome

**DOI:** 10.14740/wjon789w

**Published:** 2014-03-11

**Authors:** Andrew A. Wheeler, Michael B. Nicholl

**Affiliations:** a Department of Surgery, University of Missouri, Columbia, MO, USA; b Division of Surgical Oncology, University of Missouri, Columbia, MO, USA; c Ellis Fischel Cancer Center, University of Missouri, Columbia, MO, USA

**Keywords:** Cancer of pancreas, Surgery, Chemotherapy, Radiotherapy

## Abstract

**Background:**

Pancreatic cancer (PanCA) is predominantly diagnosed in elderly patients; nevertheless, a significant number of young patients are affected. We hypothesized more aggressive treatment of young PanCA patients would result in better overall survival (OS).

**Methods:**

A retrospective review of our institutional cancer database identified subjects for inclusion. Age 50 years was selected to stratify patients into age groups.

**Results:**

Of 309 PanCA patients, 54 (17%) were ≤ 50 years old. Exocrine cancer was the most common histology (90%). Patients ≤ 50 years old were more likely to have endocrine cancer (22% vs. 7%, P = 0.001). There was no difference in stage or curative intent surgery between age groups. Despite patients ≤ 50 years old receiving more chemotherapy (61% vs. 41%, P = 0.007) and radiotherapy (28% vs. 15%, P = 0.03), there was no difference in OS (24.1 months vs. 14.1 months, P = 0.08). When only exocrine cancers were considered, there was no difference between young and old patients regarding stage, grade, location or surgery. Exocrine cancer patients ≤ 50 years old received more chemotherapy (67% vs. 42%, P = 0.003) and radiation therapy (36% vs. 17%, P = 0.004), but there was no difference in OS.

**Conclusions:**

A substantial number of PanCA patients are ≤ 50 years old. Patients ≤ 50 years old received more treatment but did not have improved OS. Significant improvements in PanCA survival await development of new treatment strategies.

## Introduction

Pancreatic cancer (PanCA) largely affects patients of advanced age; median age at diagnosis for adenocarcinoma is 71 years [1]. Age plays an important role in treatment decision-making [2]. Few studies have investigated the impact of age on treatment and outcome of PanCA.

The currently available data suggest younger PanCA patients are more likely to receive curative intent surgery and cancer directed surgery and may have better survival [3-5]. These reports may be limited by the fact that non-operative therapy, a key component of PanCA treatment, was not reported, and by the lack of treatment detail which is common in reports derived from national population datasets [6, 7].

Although younger patients may tolerate more aggressive therapy better, patients of increasing age may undergo aggressive treatment for pancreatic tumors with good outcome [8, 9]. Pancreatic resection with good outcome has been described in octogenarians; however, other studies have demonstrated that older patients have increased rates of complications [9-11]. Older patients may have significantly worse overall health at the time of treatment which can contribute to worse outcomes [11].

Few publications focus on age-related differences in treatment provided and overall survival (OS) for PanCA. We reviewed data from our cancer data registry to exam the hypothesis that young patients with PanCA would be treated more aggressively and therefore have better prognosis. We describe differences between treatment rendered and overall outcome between patients ≤ 50 years old and those > 50 years old.

## Methods

IRB approval was obtained to query the cancer database at the Ellis Fischel Cancer Center for patients diagnosed with PanCA from 1984 to 2008. Data collected included basic demographic data, histological tumor type, site of tumor, tumor grade, tumor stage, tumor size, number of positive lymph nodes (LNs) and treatment interventions, including chemotherapy, radiation therapy and surgical treatment. Age 50 was selected to dichotomize patient groups since this was the age approximately one standard deviation below the average age of patients in the study (63 years). Survival data were updated using publically available records (social security death index). Survival comparison was based on mean survival at last follow-up. Censoring was not used.

### Statistical analysis

Demographic data including age, gender and race are presented as mean ± standard deviation. Statistical analysis was performed with SPSS^®^ 18.0, IBM^®^ Corporation (Somers, NY). Continuous data were analyzed with Student’s t test for independence, using alpha value of 0.05. Categorical data were analyzed using Chi-square test in cross-tabulation format. Statistical significance was considered at P value < 0.05 for all statistical analyses.

## Results

The mean age of the study population was 63.2 years (range 27 - 93 years). Patients > 50 years old were more likely to have an exocrine cancer than those ≤ 50 years old (93% vs. 77%, P < 0.001). The majority of patients had moderately differentiated adenocarcinoma, but there was not an association between age and tumor grade. There was no difference between age groups with respect to tumor size, tumor stage, or LN involvement. Table 1 summarizes patient demographics and tumor characteristics.

**Table 1:** Demographic and Tumor Characteristics for Pancreas Cancer Patients ≤ 50 Years Old and > 50 Years Old

	Age ≤ 50 years (%), N = 53	Age > 50 years (%), N = 254	P value
Age	44 ± 5.7	67.2 ± 9.3	< 0.001
Gender			
Male	33 (64)	143 (57)	0.372
Female	19 (37)	109 (43)	
Race			
Caucasian	34 (92)	158 (93)	0.585
African American	3 (8)	9 (5)	
Other	0 (0)	3 (2)	
Location of tumor (%)			0.541
Head of pancreas	34( 63)	146 (58)	
Body/tail of pancreas	12 (23)	59 (23)	
Unknown	7 (13)	49 (19)	
Exocrine tumor stage			0.516
I	3 (8)	16 (7)	
II	12 (30)	53 (25)	
III	8 (20)	24 (11)	
IV	17 (42)	123 (57)	

Regardless of tumor type, the rate of surgery was similar between age groups (Table 2); however, a higher number of patients ≤ 50 years old received chemotherapy as part of their treatment (62% vs. 41%, P = 0.005). Similarly, a greater number of patients ≤ 50 years old received radiation than older patients (28% vs. 15%, P < 0.03). When all tumor types were considered, OS was 24.2 months for patients ≤ 50 years old, and 13.6 months for patients > 50 years old (P = 0.06).

**Table 2:** Treatment and Outcomes in Younger Versus Older Pancreas Cancer Patients

	Age ≤ 50 (%)	Age > 50 (%)	P value
Surgery	11 (22)	51 (20)	0.911
Chemotherapy	33 (62)	104 (41)	0.005
Radiation	15 (28)	39 (15)	0.025
Number still alive	4 (8)	19 (8)	0.987
Overall survival (months)	24.2	14	0.06

Patients with exocrine tumors were significantly older than those with endocrine tumors (64.1 years vs. 54.3 years, P < 0.001) and as age increased, exocrine tumors were more common. Exocrine tumors were more likely to be located in the pancreatic head while endocrine tumors were more likely to be found in the pancreatic body or tail (62% vs. 44%, P = 0.001). Patients with endocrine tumors were more likely to undergo surgery than those with endocrine tumors (48% vs. 17%, P < 0.001), but chemotherapy treatment was not different between these groups (38% vs. 46%, P = 0.44). Of the exocrine tumor patients, 20% underwent radiation therapy, whereas no endocrine tumor patients underwent radiation therapy. Exocrine tumor patients had significantly shorter mean survival than those endocrine tumor patients (10.9 months vs. 58.7 months, P < 0.001). At last follow-up, only 4% of exocrine tumor patients were alive compared to 41% of endocrine tumor patients (P < 0.001).

**Figure 1:**
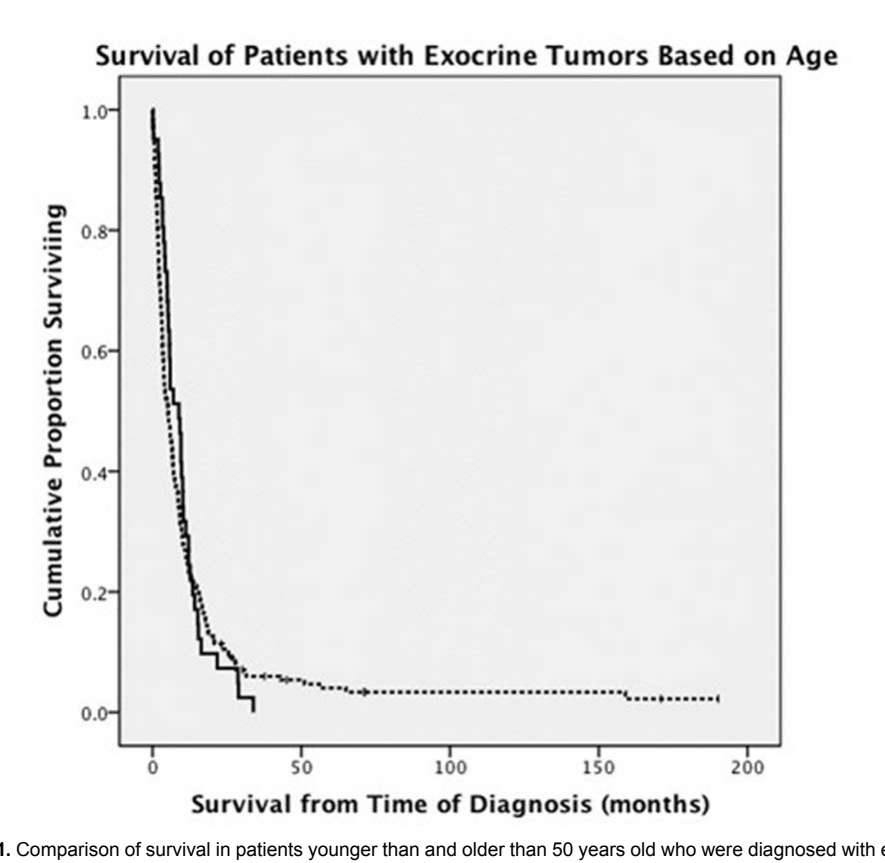
Comparison of survival in patients younger than and older than 50 years old who were diagnosed with exocrine pancreatic tumors (solid line: patients ≤ 50 years old; dashed line: patients > 50 years old).

**Figure 2:**
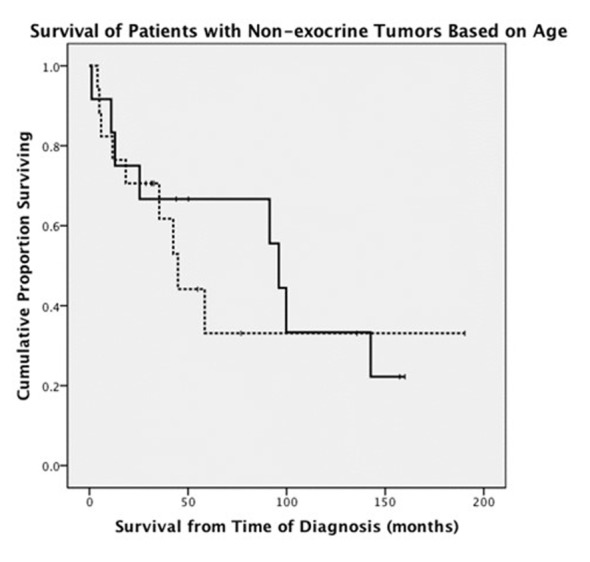
Comparison of survival in patients younger than and older than 50 years old who were diagnosed with endocrine pancreatic tumors (solid line: patients ≤ 50 years old; dashed line: patients > 50 years old).

When endocrine and exocrine cancer treatment was considered by age group, there was no difference in rate of surgery (Table 3). Exocrine cancer patients ≤ 50 years old were more likely to undergo chemotherapy (68% vs. 42%, P = 0.001) and radiation therapy (37% vs. 17%, P = 0.003). There was no difference between age groups in terms of chemotherapy used to treat endocrine cancers. OS between ages was similar in patients with exocrine tumors, 9.6 months in younger patients versus 11.2 months in older patients (Fig. 1). Endocrine cancer patients ≤ 50 years old did not have increased OS compared to older age group (Fig. 2).

**Table 3:** Age Comparison for Exocrine and Endocrine Tumors

Patient population	≤ 50 years (%)	> 50 years (%)	P value
Exocrine	41 (15)	237 (85)	
Endocrine	12 (41)	17 (59)	
Age			
Exocrine	45 ± 4.7	67.4 ± 9.3	< 0.001
Endocrine	40.7 ± 7.6	64 ± 8.2	< 0.001
Location of tumor			
Exocrine			0.24
Head of pancreas	30 (73)	142 (60)	
Body/tail of pancreas	7 (17)	51 (21)	
Unknown	4 (10)	44 (19)	
Endocrine			0.84
Head of pancreas	4 (33)	4 (24)	
Body/tail of pancreas	5 (39)	8 (47)	
Unknown	3 (38)	5 (29)	
Surgery performed			
Exocrine	7 (17)	41 (17)	0.97
Endocrine	4 (33)	10 (59)	0.23
Chemotherapy			
Exocrine	28 (68)	98 (42)	0.001
Endocrine	5 (42)	6 (35)	0.73
Radiation therapy			
Exocrine	15 (37)	39 (17)	0.003
Endocrine	0	0	
Number still alive			
Exocrine	0 (0)	11 (5)	0.16
Endocrine	4 (33)	8 (47)	0.46
Overall survival (months)			
Exocrine	9.6	11.2	0.64
Endocrine	74.3	47.6	0.19

## Discussion

Successful treatment of an aggressive cancer warrants an aggressive approach [12]. Aggressive treatment can include multimodality therapy, more intensive therapy, or more invasive therapy. Surgical treatment of PanCA improves survival and post-surgical adjuvant therapy further modestly extends this result [13-16]. Completing PanCA treatment regimens is difficult but important to enhance survival. Higher utilization of therapy and better ability to tolerate intensive therapy would be expected to correlate with improved outcome. Young patients, in part because of fewer comorbidities, should be better able to withstand aggressive treatment.

Age is associated with the likelihood of completing therapy, but the role that age plays in PanCA survival is more complex [3-5, 17-19]. Younger patients are more likely to undergo surgical resection as well as adjuvant therapy [3-5, 20, 21]. Finlayson et al demonstrated that while older patients undergoing surgery had more comorbidities and a higher complication rate, 5-year survival did not differ from younger patients [10]. Others have shown increased survival in younger surgical patients [18]. Studies are also conflicting with regard to the effects of chemotherapy based on age [22-24]. These studies lack a uniform definition for old age. There is no agreement as to which age constitutes old and this is reflected in the literature. PanCA patients from ages 65 years to 80 years have been categorized as old [25].

We examined the distribution of age among PanCA patients to define young and old age for this study. Increasing comorbid conditions and worsening performance status with age are relative contraindications to aggressive treatment in older patients. We expected to find age-related survival differences due at least in part to differences in treatment administered. While younger patients in our study received more treatment for PanCA, they did not have improved OS compared to the older patients. As would be expected, we found exocrine PanCA was treated differently than endocrine PanCA [18]. Even among the PanCA subtypes, no OS difference was seen. Our data do not support that increased therapy in younger patients leads to improved outcomes.

The Ellis Fischel Cancer Center is the oldest cancer center west of the Mississippi River and opened its doors as the central hub of the Missouri state cancer program in 1949 [26]. Missouri cancer data are not captured by the SEER program; therefore, data reported in this study are unique from studies previously published from the SEER database. The longitudinal nature of this study can be considered both a strength and weakness. Data were collected from patients across many eras of PanCA treatment. None of the more recent innovations such as neoadjuvant therapy or FOLFIRINOX were available to patients in this study. Other limitations of the study include the small study size, particularly in the younger age group; therefore, non-significant findings should be interpreted cautiously.

We believe improvements in PanCA treatment rely on development of new therapies and techniques rather than more aggressive use of the currently available modalities. As such, neoadjuvant therapy is currently being widely studied both as a means to downstage tumors to improve chance of resectability as well as determine patients most likely to benefit from pancreatic resection [27-29]. Additionally, post-operative complications after pancreatic resection do not prevent adjuvant chemotherapy administration [30]. With newer and more aggressive therapies to augment surgical resection, survival may be improved. Promising new therapies include the addition of molecular target agents with currently used cytotoxic agents as well as immunotherapy combined with cytotoxic therapy [31-33].

Review of PanCA patients treated over the last quarter century at our cancer institute shows those ≤ 50 years old have a different histologic profile than those patients > 50 years old, receive more treatment, but do not have better survival. Many problems with treating PanCA still exist. Too many patient are not candidates for treatment or do not receive treatment, the treatment options are harsh, treatment complications may prohibit completion of therapy and the treatments are not particularly effective. Thus, despite increased use of aggressive therapy in younger patients, outcomes are not better. Improvements in PanCA are dependent upon the future development of more effective therapy than bringing more patients to receive the currently available therapy.
